# *DNAJC6* Mutations Disrupt Dopamine Homeostasis in Juvenile Parkinsonism‐Dystonia

**DOI:** 10.1002/mds.28063

**Published:** 2020-05-30

**Authors:** Joanne Ng, Elisenda Cortès‐Saladelafont, Lucia Abela, Pichet Termsarasab, Kshitij Mankad, Sniya Sudhakar, Kathleen M. Gorman, Simon J.R. Heales, Simon Pope, Lorenzo Biassoni, Barbara Csányi, John Cain, Karl Rakshi, Helen Coutts, Sandeep Jayawant, Rosalind Jefferson, Deborah Hughes, Àngels García‐Cazorla, Detelina Grozeva, F. Lucy Raymond, Belén Pérez‐Dueñas, Christian De Goede, Toni S. Pearson, Esther Meyer, Manju A. Kurian

**Affiliations:** ^1^ Molecular Neurosciences, Developmental Neurosciences Programme UCL Great Ormond Street Institute of Child Health London United Kingdom; ^2^ Gene Transfer Technology Group UCL Institute for Women's Health London United Kingdom; ^3^ Department of Neurology Icahn School of Medicine at Mount Sinai New York USA; ^4^ Division of Neurology, Department of Medicine, Faculty of Medicine Ramathibodi Hospital Mahidol University Bangkok Thailand; ^5^ Department of Radiology Great Ormond Street Hospital for Children NHS Foundation Trust London United Kingdom; ^6^ Department of Neurology Great Ormond Street Hospital for Children NHS Foundation Trust London United Kingdom; ^7^ Neurometabolic Unit National Hospital for Neurology and Neurosurgery London United Kingdom; ^8^ Department of Nuclear Medicine and Imaging Lancashire Teaching Hospitals, NHS Foundation Trust Preston United Kingdom; ^9^ Department of Paediatrics East Lancashire Hospital NHS Trust Lancashire United Kingdom; ^10^ Department of Paediatric Neurology John Radcliffe Hospital, Oxford University, NHS Foundation Trust London United Kingdom; ^11^ Department of Paediatrics Royal Berkshire Hospital, NHS Foundation Trust Reading United Kingdom; ^12^ Molecular Neuroscience and Reta Lila Weston Laboratories Institute of Neurology Queen Square London United Kingdom; ^13^ Department of Neurology Neurometabolic Unit and CIBERER Hospital Sant Joan de Déu, Esplugues de Llobregat Barcelona Spain; ^14^ Medical Genetics Cambridge Institute for Medical Research, University of Cambridge Cambridge United Kingdom; ^15^ UK10K Project, Wellcome Trust Sanger Institute Hinxton Cambridge United Kingdom; ^16^ Hospital Vall d'Hebron, Institut de Recerca (VHIR) Barcelona Spain; ^17^ Department of Paediatric Neurology Royal Preston Hospital, Lancashire Teaching Hospitals, NHS Foundation Trust London United Kingdom; ^18^ Department of Neurology Washington University School of Medicine St. Louis Missouri USA

**Keywords:** auxilin, *DNAJC6*, dopamine, dystonia, parkinsonism

## Abstract

**Background:**

Juvenile forms of parkinsonism are rare conditions with onset of bradykinesia, tremor and rigidity before the age of 21 years. These atypical presentations commonly have a genetic aetiology, highlighting important insights into underlying pathophysiology. Genetic defects may affect key proteins of the endocytic pathway and clathrin‐mediated endocytosis (CME), as in DNAJC6‐related juvenile parkinsonism.

**Objective:**

To report on a new patient cohort with juvenile‐onset *DNAJC6* parkinsonism‐dystonia and determine the functional consequences on auxilin and dopamine homeostasis.

**Methods:**

Twenty‐five children with juvenile parkinsonism were identified from a research cohort of patients with undiagnosed pediatric movement disorders. Molecular genetic investigations included autozygosity mapping studies and whole‐exome sequencing. Patient fibroblasts and CSF were analyzed for auxilin, cyclin G–associated kinase and synaptic proteins.

**Results:**

We identified 6 patients harboring previously unreported, homozygous nonsense *DNAJC6* mutations. All presented with neurodevelopmental delay in infancy, progressive parkinsonism, and neurological regression in childhood. ^123^I‐FP‐CIT SPECT (DaTScan) was performed in 3 patients and demonstrated reduced or absent tracer uptake in the basal ganglia. CSF neurotransmitter analysis revealed an isolated reduction of homovanillic acid. Auxilin levels were significantly reduced in both patient fibroblasts and CSF. Cyclin G–associated kinase levels in CSF were significantly increased, whereas a number of presynaptic dopaminergic proteins were reduced.

**Conclusions:**

*DNAJC6* is an emerging cause of recessive juvenile parkinsonism‐dystonia. *DNAJC6* encodes the cochaperone protein auxilin, involved in CME of synaptic vesicles. The observed dopamine dyshomeostasis in patients is likely to be multifactorial, secondary to auxilin deficiency and/or neurodegeneration. Increased patient CSF cyclin G–associated kinase, in tandem with reduced auxilin levels, suggests a possible compensatory role of cyclin G–associated kinase, as observed in the auxilin knockout mouse. *DNAJC6* parkinsonism‐dystonia should be considered as a differential diagnosis for pediatric neurotransmitter disorders associated with low homovanillic acid levels. Future research in elucidating disease pathogenesis will aid the development of better treatments for this pharmacoresistant disorder. © 2020 The Authors. *Movement Disorders* published by Wiley Periodicals, Inc. on behalf of International Parkinson and Movement Disorder Society.

Classical Parkinson's disease (PD) is an age‐related neurodegenerative disorder, mainly affecting adults aged >50 years. Patients typically present with resting tremor, bradykinesia, rigidity, and postural instability. To date, a number of early‐onset genetic forms of PD (*Parkin*, *PINK1*, and *DJ‐1*)^1^, ^2^, ^3^ and complex parkinsonism syndromes (*ATP13A2*, *PLA2G6*, *FBXO7*, *SLC6A3*, *SLC39A14*, and *PANK2*) have been described.^4^, ^5^, ^6^, ^7^, ^8^, ^9^ Importantly, the study of such monogenic forms of disease have provided significant insight into the pathogenic mechanisms underlying sporadic PD.^10^ More recently, two genes, namely *SYNJ1* 11 and *DNAJC6*,^12^, ^13^, ^14^ encoding proteins involved in postendocytotic recycling of synaptic vesicles, have been identified in early‐onset parkinsonism.

In this study, we report on 6 children from three families, presenting with juvenile parkinsonism‐dystonia associated with novel, biallelic *DNAJC6* mutations. We delineate their clinical phenotype, neuroimaging features (including ^123^I‐FP‐CIT single‐photon emission computed tomography [SPECT; DaTScan]) and pattern of cerebrospinal fluid (CSF) neurotransmitter metabolites. Furthermore, we utilized patient fibroblasts and CSF to investigate secondary effects on auxilin, cyclin G–associated kinase (GAK), and dopaminergic proteins.

## Materials and Methods

### Subject Recruitment:

A cohort of 232 children with undiagnosed movement disorders were recruited for research between 2012 and 2016 at UCL Great Ormond Street–Institute of Child Health (London, UK). A subgroup of 25 patients were identified with juvenile parkinsonism, defined as onset of bradykinesia before 21 years of age, and at least one of the following signs: resting tremor, rigidity, and postural instability. All patients had detailed clinical assessment, undertaken by a movement disorder specialist. Review of (1) the clinical history, (2) features on neuroimaging, and (3) video recordings of the movement disorder at different time points was undertaken. Written informed consent was obtained from participating families, and the study was approved by the local ethics committees (Reference 13/LO/0168).

### Diagnostic CSF Neurotransmitter Analysis:

In order to rule out a primary neurotransmitter disorder, where possible, patients had a routine diagnostic lumbar puncture for CSF neurotransmitter analysis. Using standardized protocols,^15^ CSF samples were collected, snap frozen in liquid nitrogen, and stored at –80°C. Analysis was undertaken using high‐pressure liquid chromatography (HPLC) with electrochemical detection and reversed‐phase column.^15^ Seven anonymized control pediatric CSF samples (with normal CSF neurotransmitter profiles) were obtained from the Neurometabolic Laboratory (National Hospital for Neurology and Neurosurgery, London, UK). All samples were processed and stored in accordance with the UK Royal College of Pathologists guidelines.

### Molecular Genetic Investigation:

From the subgroup of 25 patients with juvenile parkinsonism (16 singletons and 9 familial cases from 3 kindreds), we prioritized two consanguineous families (Family A, 3 affected children; Family B, 2 affected children) for initial analysis. These families were investigated using an autozygosity mapping approach, given that the affected children were phenotypically similar and both families originated from the same region in Pakistan. Single‐nucleotide polymorphism (SNP) genotyping was performed as previously described.^16^ In addition, whole‐exome sequencing (WES) was performed for 2 children (A:III‐1 and B:IV‐2) by UCL Genomics (average WES coverage as previously reported^17^), with an average *DNAJC6* coverage of 30×, with minimum coverage 10× for 82% of the gene. WES data were probed for putative disease‐causing *DNAJC6* mutations in the remaining 20 cases (16 sporadic patients, 4 familial cases from a single kindred). This was undertaken through UCL Genomics (8 patients) and Wellcome Trust Sanger Institute (12 patients) within the Wellcome Trust UK10K Rare Diseases project, as previously reported.^18^ For patients where *DNAJC6* mutations were identified, whole‐exome data were also probed for other genes associated with early‐onset dystonia‐parkinsonism (Table 1).

**Table 1:** Clinical characteristics of patients with *DNAJC6* mutations from this cohort and literature

	Pat. 1	Pat. 2	Pat. 3	Pat. 4	Pat. 5	Pat. 6	Previously Described *DNAJC6*‐Related Childhood Parkinsonism Dystonia Phenotypes in the Literature							Previously Described *DNAJC6*‐Related Early Onset PD in the Literature				
	A‐III:1	A‐III:4	A‐III:5	B‐IV:2	B‐IV:4	C	II‐2^12^	II‐4	402^13^	502	504	505	42029^21^	GPS313^14^	GPS314	PAL50	PAL54	BR‐2652
Presenting age (years)/sex	20/F	12/M	10/M	28/F	19/F	18/F	18/M	13/M	44/F	24/F	31/F	17/M	10/F	48/M	44/F	62/M	46/F	57/M
Consanguinity	Y	Y	Y	Y	Y	Y	Y	Y	Y	Y	Y	Y	Y	N	N	N	N	N
Country of origin	PK	PK	PK	PK	PK	PR	PL	PL	TR	TR	TR	TR	YM	NL	NL	BR	BR	BR
Onset of parkinsonism (y)	11	10	9	13	7	10	7	11	10	11	10	10	10	21	29	42	31	24
Parkinsonism at presentation	Y	Y	Y	Y	Y	Y	Y	Y	Y	Y	Y	Y	N	Y	N	Y	Y	N
Early development	D	D	D	D	D	D	N	N	N	D	D	D	N	N	N	N	N	N
Bradykinesia	+	+	+	+	+	+	+	+	+	+	+	+	+	+	+	+	+	+
Tremor	+	+	‐	+	‐	+	+	+	+	+	+	+	+	+	+	+	+	+
Rigidity	+	+	+	+	+	+	+	+	+	+	+	+	+	+	+	+	+	+
Hypomimia	+	+	+	+	‐	+	+	+	+	+	+	+	+	+	+	+	+	+
Postural instability	+	+	+/‐	+	+	+	+	+	+	+	+	+	+	+	+	+	+	+
Dystonic posturing	+	+	‐	+	‐	+	‐	‐	+	+	+	+	‐	‐	‐	‐	‐	‐
Dysarthria	Anarthric	+	+/‐	Anarthric	NR	+	+	+	+	+	+	+	‐	‐	‐	‐	‐	‐
Eye movements abnormal	+	‐	‐	NR	‐	‐	‐	+	NR	NR	NR	NR	NR	NR	NR	NR	NR	NR
Loss of ambulation (y)	13	Unsteady gait	‐	13	10	15	18	13	39	21‐26	31	20‐25	12	Wheelchair bound	‐	‐	‐	‐
Motor fluctuations	+	‐	‐	‐	‐	+	NR	NR	NR	NR	NR	NR	NR	‐	‐	+	+	+
Seizures	‐	‐	‐	+	‐	+	‐	‐	+	‐	+	+	+	‐	‐	‐	‐	‐
Cognition	CI	CI	CI	CI	CI	CI	N	N	CI	CI	CI	CI	CI	NR	NR	CI	NR	NR
Spasticity	‐	‐	‐	‐	‐	‐	‐	‐	+	+	+	+	+	‐	‐	‐	‐	‐
Psychiatric features	Ax	Ax, PsB	‐	Ax	‐	AD, AgB	NR	NR	‐	Psy after l‐dopa, AgB, Hall	‐	‐	Psy, visual and auditory Hall	NR	Psy	NR	NR	NR
Other clinical features	MC, hypothyroid	MC, SD	MC	SD	SD	Mild BP	NR	NR	Scol	Mcl	Negative Mcl, Scol	‐	‐	NR	NR	NR	NR	NR
Brain imaging (MRI/CT) (Atrophy)	Perisylvian, cerebellar	N	N	Mild, generalized	Mild generalized, cerebellar	Mild, generalized, thin corpus callosum	N	N	Generalized	N	N	N	N	N	N	N	N	N
^123^I‐FP‐CIT SPECT (DaTScan™) (Uptake in BG)	Absent	Reduced	Not performed	Not performed	Absent	Not performed	NR	NR	NR	NR	NR	NR	NR	NR	NR	NR	NR	NR
Genetics																		
*DNAJC6* mutation	c.766 C>T	c.766 C>T	c.766 C>T	c.766 C>T	c.766 C>T	c.2416 C>T	c.801‐2A>G	c.801‐2A>G	c.2200 C>T	c.2200C>T	c.2200 C>T	c.2200 C>T	c.2365 C>T	c.2779 A>G	c.2779 A>G	c.2223 A>T	c.2223 A>T	c.2038+3 A>G and c.1468+83del/‐
Protein change	R256*	R256*	R256*	R256*	R256*	R806*			Q734X	Q734X	Q734X	Q734X	Gln789*	R927G	R927G	Thr741*	Thr741*	
Variants in other PD genes^a^	N	N	N	N	N	N	NR	NR	NR	NR	NR	NR	NR	NR	NR	NR	NR	NR
Response to l‐dopa/carbidopa (maximum dose)	Unsustained (150 mg/d)	Not tried	Not tried	Unsustained (5.5 mg/kg/d)	No response (10 mg/kg/d)	Some response (200 mg/d)	NR	No response	Good	Good	Therapy refused	Good	Mild	Good, dosages limited by psychiatric side effects	Good, dosages limited by psychiatric side effects	Good	Good	Good

a *ATP13A2*, *ATP1A3*, *ATP7B*, *ATXN2* (*SCA2*), *ATXN3* (*SCA3*), *C19orf12*, *C9orf72*, *CSF1R*, *DCTN1*, *DDC*, *DJ1* (*PARK7*), *EIF4G1*, *FBXO7*, *FMR1*, *FTL*, *GCH1*, *GIGYF2*, *GRN*, *HTRA2*, *HTT*, *LRRK2*, *MAPT*, *ND4*, *NPC1*, *PANK2*, *PARKIN* (*PARK2*), *PINK1*, *PITX3*, *PLA2G6*, *POLG*, *PPP2R2B*, *PRKRA*, *PTS*, *QDPR*, *SLC6A3*, *SLC18A2*, *SLC30A10*, *SNCA*, *SPATACSIN* (*SPG11*), *SR* (*SNCG*), *SYNJ1*, *TAF1*, *TBP*, *TH*, and *UCHL1*.

Y, years of age; F, female; M, male; N, no; Y, yes; NR, not reported; PK, Pakistan; PL, Palestine; TR, Turkey; YM, Yemen; NL, Netherlands; BR, Brasil; Cl, cognitive impairment; AD, attention deficit; AgB, aggressive behavior; Ax, anxiety; PsB, perseveration behavior; Psy, psychosis; Hall, hallucinations; MC, microcephaly; SD, sleep disturbance; BP, behavioral problems; Mcl, myoclonus; Scol, scoliosis; BG, basal ganglia.

#### Direct Sanger Sequencing

Sanger sequencing was used to confirm variants identified on WES and to establish familial segregation. A genomic *DNAJC6* sequence (Ensembl transcript: ENST00000371069; NCBI reference sequence: NM_001256864) was utilized to design primers, using Primer3 software (http://bioinfo.ut.ee/primer3/). Primers and polymerase chain reaction (PCR) amplification conditions are available on request. PCR products were cleaned up with MicroCLEAN (Web Scientific) and directly sequenced using Big Dye Terminator Cycle Sequencing System (Applied Biosystems Inc., Foster City, CA). Sequencing reactions were run on an ABI PRISM 3730 DNA Analyzer (Applied Biosystems Inc.) and analyzed with Chromas (http://www.technelysium.com.au/chromas.html).

### Fibroblast and CSF Immunoblotting:

Methods to assess protein expression in patient fibroblasts were as previously reported.^17^ In brief, primary fibroblast lines were cultured from skin biopsies taken from Patients A‐III:1 and B‐IV:4 (c.766C>T; p.R256*) and 2 age‐matched healthy donor controls. Antibodies for auxilin and GAK (gift from Professor Green, National Institutes of Health, Washington, DC) and glyceraldehyde 3‐phosphate dehydrogenase (GAPDH) horseradish peroxidase (HRP) conjugate (Cell Signaling Technology, Inc., Danvers, MA) were used.

Patients A‐III:1, A‐III:4, and B‐IV:4 underwent lumbar puncture for diagnostic CSF neurotransmitter analysis, and three CSF aliquots were snap frozen and stored at −80°C. Seven age‐matched control CSF samples were identified, from subjects without movement disorders on no medication. Patient and control CSF samples were immunoblotted for auxilin, GAK, and dopaminergic proteins as described previously.^19^ CSF protein was probed with the following antibodies: auxilin, GAK, tyrosine hydroxylase (MilliporeSigma, Burlington, MA), dopamine receptor 2 (MilliporeSigma), dopamine transporter (MilliporeSigma), vesicular monoamine transporter 2 (Santa Cruz Biotechnology, Inc., Santa Cruz, CA), and transferrin (Santa Cruz Biotechnology, Inc) as the loading control. Relative protein levels were quantified using ImageJ software (National Institutes of Health, Bethesda, MD) and normalized to the loading control and the mean percentage of optical densitometry of three replicates analyzed with standard error of the mean.

### Statistical Analysis

Statistical analysis was performed using Prism software (version 8; GraphPad Software Inc., La Jolla, CA), with data tested for Gaussian distribution and compared by the Student *t* test.

### Data Availability Statement:

All clinical and experimental data relevant to this study are contained within the article. For Families A, B, and C, there is no ethical approval in place for deposition of whole‐exome sequencing genomic data into a public repository. Genomic data from UK10K are available at the EGA European Genomen Phenome Archive (https://www.ebi.ac.uk/ega/home), EGAS00001000128(UK10K RARE FIND). Details of statistical analysis can be shared upon request.

## Results

### Patient Cohort

**Figure 1:**
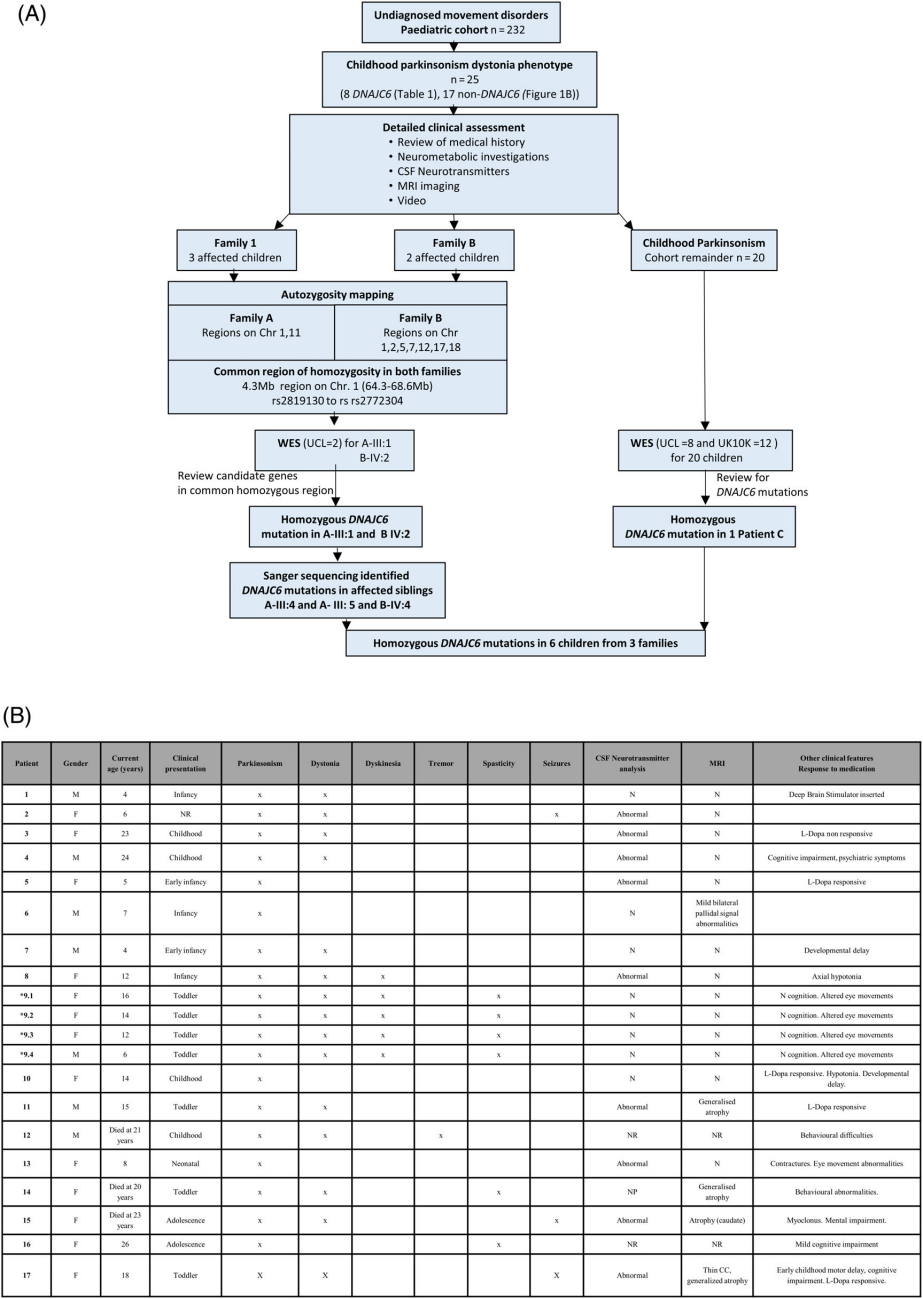
Juvenile parkinsonism cohort: clinical features and molecular genetic investigation. (**A**) Flowchart demonstrating the pathway of molecular genetic investigations in a subcohort of 25 children with juvenile parkinsonism. (**B**) Clinical characteristics of 20 children from 17 families. Early infancy <3 months; infancy 3 to 12 months; toddler 12 to 24 months; childhood 2 to 13 years; adolescence 13 to 18 years. *Consanguineous family. M, male; F, female; N, normal; NP, not performed; NR, not reported. [Color figure can be viewed at wileyonlinelibrary.com]

A total of 232 children were referred with undiagnosed movement disorders for genetic research (Fig. 1A). Of these, 25 children (10.7%) had juvenile parkinsonism, 16 females and 9 males with a current median age of 14 years (range, 4–28). Fourteen of 25 had additional neurological features, including dystonia (14 patients), developmental delay/learning difficulties (14 patients), and seizures (4 patients; Fig. 1B).

### Molecular Genetic Investigations

**Figure 2:**
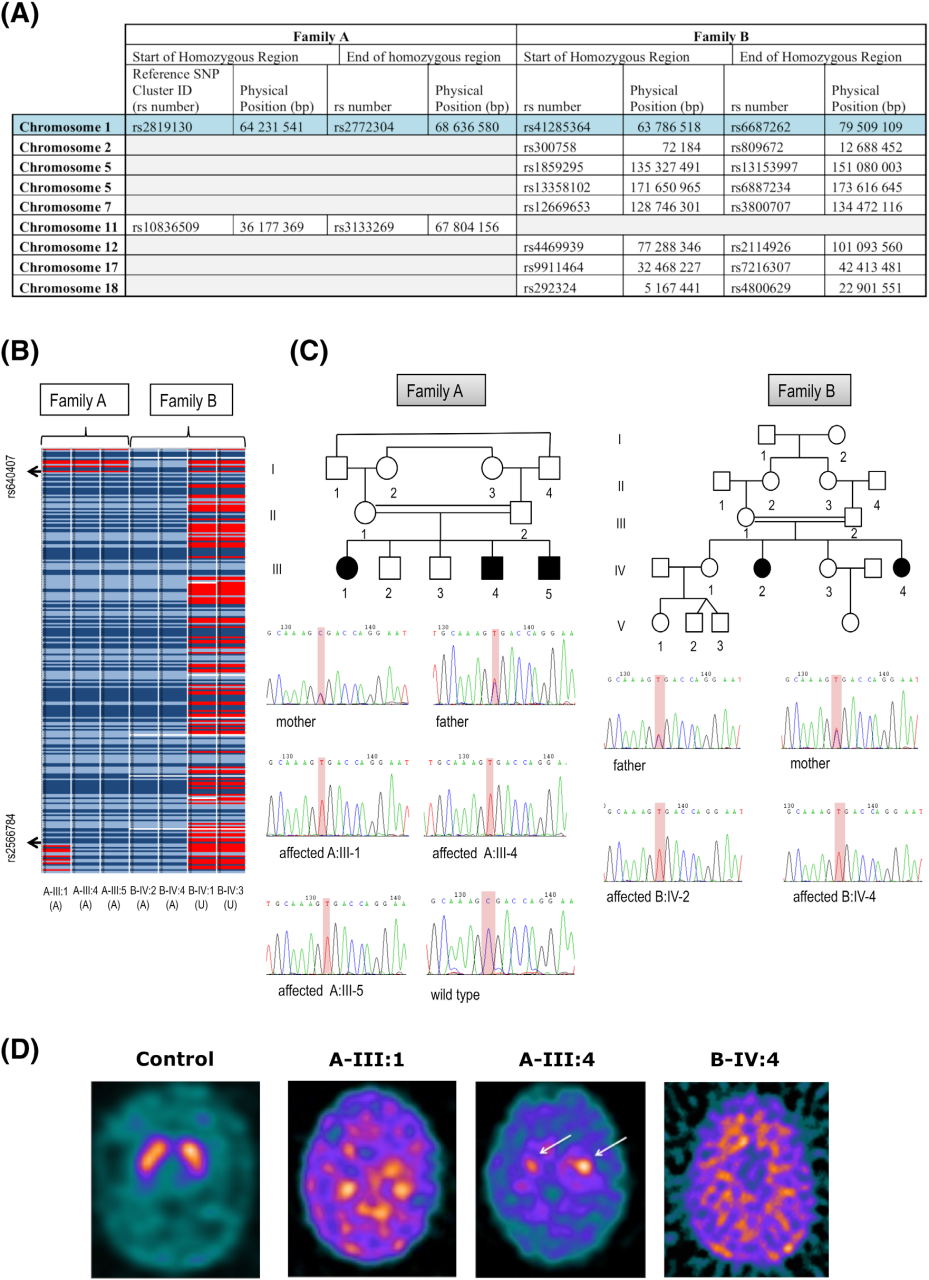
Molecular genetic investigations and DaTSCAN imaging. (**A**) Family A and B SNP array results showing homozygous regions detected. For each chromosome, the start and end point is specified using the Reference SNP Cluster ID (rs number) and physical position. (**B**) Homozygous SNPs are represented in light blue (AA) and dark blue (BB), heterozygous SNPs in red (AB), and “no calls” in white. (**C**) Sanger Sequencing confirms a homozygous *DNAJC6* mutation, c.766C > T (p.R256*), in all affected children of Family A (A‐III:1, A‐III:4, and A‐III:5) and Family B (B‐IV:2, B‐IV:4). Parents are heterozygous carriers. (**D**) I‐123‐DaTSCAN™ with SPECT imaging in a control subject, Patient A‐III:1 (19 years 3 months), Patient A‐III:4 (11 years 4 months), and Patient B‐IV:4 (17 years). In Patients A‐III:1 and B‐IV:4, DaTSCAN findings indicate virtually complete absence of tracer uptake in the basal ganglia, with very high background activity, suggesting loss of presynaptic dopaminergic terminals, whereas Patient A‐III:4 showed significantly reduced, albeit still visible, uptake in the head of caudate (left better than right, white arrows). [Color figure can be viewed at wileyonlinelibrary.com]

Families A and B (Fig. 1A) were prioritized for autozygosity mapping studies. SNP genotyping revealed a 4.33‐Mb region of common homozygosity in both families on chromosome 1, between rs640407 (64,267,606 base pairs [bp]) and rs2566784 (68,602,735 bp; Fig. 2A,B). This region showed a common haplotype in all affected children whereas unaffected siblings had a different haplotype. It was therefore considered to be the likely disease locus (Fig. 2B).

WES performed in Patients A‐III:1 and B‐IV:2 revealed 23,365 and 23,549 variants, respectively. Given familial consanguinity and the autozygosity mapping results, targeted analysis for recessive pathogenic variants within the putative disease locus on chromosome 1 was undertaken. A single homozygous nonsense variant c.766C>T (p.R256*) in *DNAJC6* (Chr1, 65,248,219–65,415,869) was identified both in A‐III:1 and B‐IV:2 on WES, located within the common region of homozygosity. No other pathogenic changes in previously reported genes causing juvenile parkinsonism‐dystonia phenotypes were identified from the WES data. Direct Sanger sequencing confirmed the homozygous c.766C>T mutation in all 5 affected patients, and familial segregation studies revealed that all parents were obligate carriers in both kindreds, with unaffected siblings either wild type or heterozygous for the identified variant (Fig. 2C). WES/whole‐genome sequencing data for the remainder of the parkinsonism‐dystonia cohort (n = 20) was interrogated for *DNAJC6* mutations. This led to the identification of a homozygous nonsense variant (c.2416C>T) in a sixth unrelated patient (Patient C), which was confirmed on Sanger sequencing.

### Delineation of the Clinical Phenotype of *DNAJC6* Patients

#### Family A (3 Affected Patients)

Patients A‐III:1, A‐III:4, and A‐III:5 are 3 affected children born to first‐cousin parents, currently 20, 12, and 10 years old (Table 1). Two other brothers (A‐III:2 and A‐III:3), aged 17 and 15 years, have mild learning difficulties without evidence of a movement disorder. The paternal grandfather was diagnosed with PD in his fifties.

All 3 children were born at term after an uneventful antenatal period. Microcephaly was evident at birth (head circumference: <0.4th centile), but nonprogressive over time. All siblings had early neurodevelopmental delay and moderate learning difficulties.

**Figure 3:**
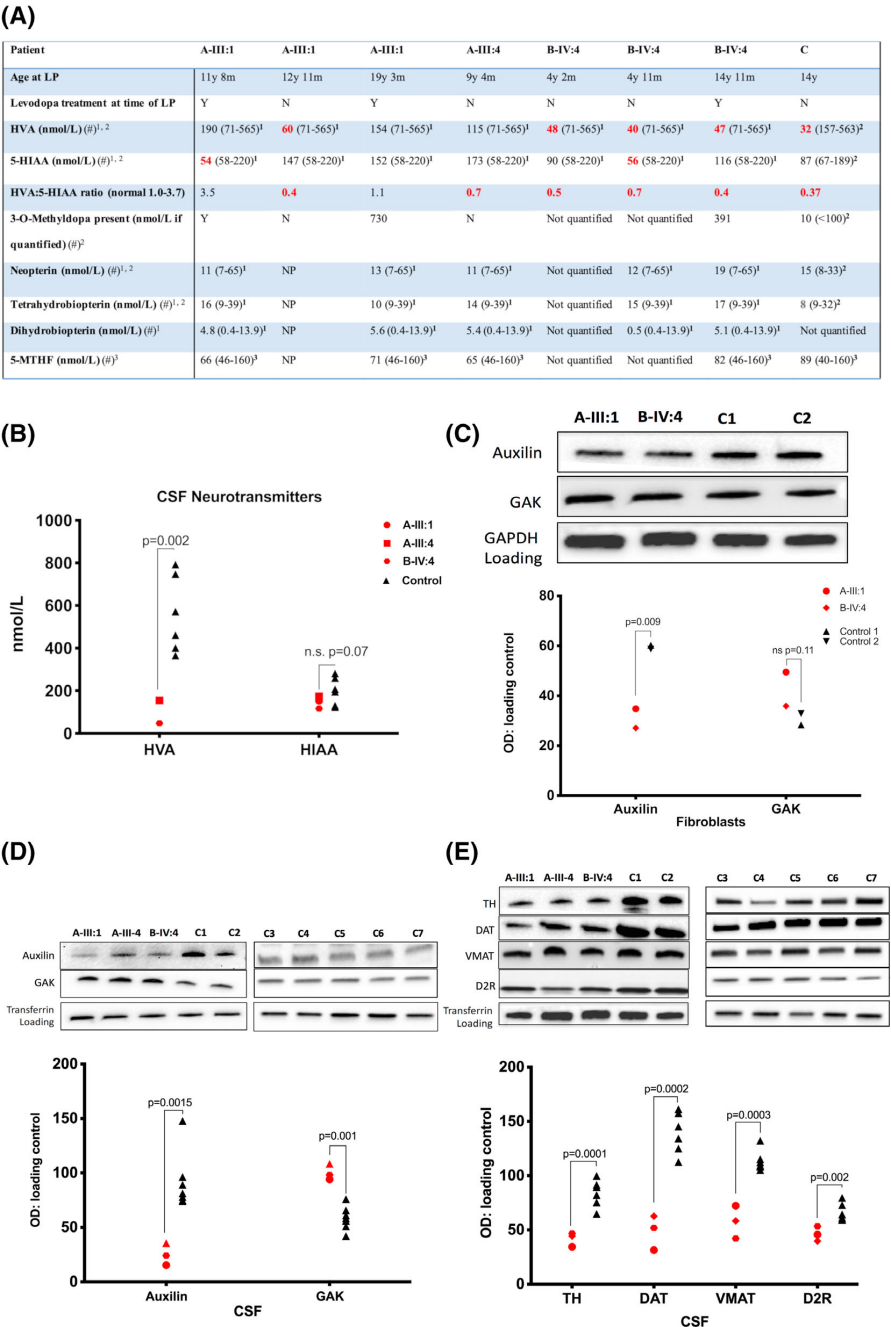
CSF neurotransmitter analysis and patient fibroblast and CSF immunoblotting. (**A**) CSF neurotransmitter analysis. Age‐related reference ranges indicated in brackets after each value. Red: abnormal result. Gray: borderline result. Symbol (“#”) indicates reference range: ^1^Keith Hyland, Robert A.H. Surtees, et al. Pediatr Res 1993;34:10–14; ^2^Keith Hyland, Future Neurol 2006;1:593–603; ^3^Surtees R, Hyland K. Biochem Med Metab Biol 1990;44:192–199. (**B**) Scatterplot of CSF HVA and 5‐HIAA levels (nmol/L) measured by high performance liquid chromatography (patient = red shapes, control = black triangles). Medication at time of CSF sampling: A‐III‐1: co‐careldopa, melatonin, glycopyronium; A‐III‐4: none; B‐IV‐4: l‐dopa, pyridoxine; Control 1: none; Control 2: none. Immunoblot of auxilin and GAK in patient fibroblasts (**C**) and CSF (**D**) compared to controls. (**E**) Immunoblot of patient CSF for TH, DAT, VMAT, and D2R protein levels measured compared to controls. Graphs show mean protein percent optical density (OD) normalized to loading control in patients (red) and controls (black). LP, lumbar puncture; y, years; m, months; 5‐MTHF, 5‐methyltetrahydrofolate; NP, not performed. [Color figure can be viewed at wileyonlinelibrary.com]

A‐III:1 is the eldest daughter, aged 20 years. She presented at 10 years, with a 6‐week history of feeding difficulties, vomiting, and weight loss. Over time, she developed fever, unsteady gait, facial asymmetry, left‐sided tremor, and generalized seizures and was diagnosed with an encephalitis of uncertain etiology. She recovered from this acute illness, but subsequently had progressive bradykinesia, with tremor and rigidity, and loss of independent ambulation at 13 years, associated with cognitive decline. She is now wheelchair dependent, with generalised cogwheel‐rigidity, severe bradykinesia, multiple limb contractures and emotional lability (Video 1). She also has severe gut dysmotility, with recurrent vomiting, and required a gastrostomy for deteriorating bulbar dysfunction. CSF neurotransmitter analysis (age 11 years), while on levodopa therapy, revealed an isolated low 5‐hydroxyindoleacetic acid (5‐HIAA; Fig. 3A). At 12 years 11 months, when off l‐dopa, CSF HVA, and HVA:5‐HIAA ratio were low. Brain MRI showed evidence of right‐sided atrophy of the perisylvian region and right cerebellum by 19 years of age (Supporting Information Fig. S1). At 19 years, ^123^I‐FP‐CIT SPECT (DaTScan) showed absent uptake in the basal ganglia when compared to normal subjects (Fig. 2D). At this stage, while on l‐dopa treatment, her CSF HVA levels normalized (Fig. 3A). Her condition is refractory to medical treatment, with no clinical response to trihexyphenidyl, benzhexol, procyclidine, clobazam, rotigotine, and apomorphine. l‐dopa has proven difficult to titrate because of marked drug sensitivity. She experiences an improvement in motor function and speech 30 minutes postdose, after which she returns to the *off* state. l‐dopa dosages >150 mg/d have resulted in drug‐related dyskinesias.

Her two brothers (A‐III:4 and A‐III:5) presented with fine motor difficulties at 8 years of age. They subsequently developed positional tremor, upper limb dystonic posturing, hypophonia, hypomimia, bradykinesia, cogwheel rigidity, and postural instability over 12 months (Videos 2 and 3). Like their sister, both have gastrointestinal complications with sialorrhea, recurrent vomiting, and feeding difficulties, necessitating gastrostomy insertion. A‐III:4 is currently 12 years old and suffers from anxiety and perseveration. His CSF‐HVA levels are at the lower limit of normal, with a low HVA:5‐HIAA ratio <1.0 (Fig. 3A). MRI brain scan was normal. ^123^I‐FP‐CIT SPECT (DaTScan) at 11 years showed profound reduction in tracer uptake in the basal ganglia (Fig. 2D). Both boys responded to treatment with transdermal rotigotine and oral trihexyphenidyl, but with increasing doses, both experienced dyskinesias, necessitating dose reduction.

#### Family B (2 Affected Patients)

Patients B‐IV:2 and B‐IV:4 are 2 affected girls, born to first‐cousin parents, and currently 28 and 19 years old. Both were born uneventfully following a normal pregnancy, presenting with early feeding difficulties, hypotonia, and delayed milestones by 6 months old. Both made slow developmental progress, achieving independent ambulation and spoken language by 3 years of age.

B‐IV:2 presented at 9 years with generalized seizures that stabilized with lamotrigine therapy. From 13 years of age, motor and cognitive deterioration ensued, with onset of parkinsonism and loss of speech and ambulation. She experienced anxiety and recurrence of seizures. She has severe antecollis, hypomimia, tremor, generalised cogwheel rigidity, bradykinesia, and positive glabellar tap (Video 4). MRI was normal until 18 years, after which there was radiological evidence of mild generalized atrophy. Several medications were tried without clinical benefit, including l‐dopa (maximum, 10 mg/kg/d), selegiline, rotigotine, and trihexyphenidyl. The younger sibling, B‐IV:4, presented at 7 years with gait deterioration, bradykinesia, and cogwheel rigidity. She lost independent ambulation and speech by 10 years (Video 5). She has developed dystonic posturing, bulbar dysfunction (necessitating gastrostomy), and a disrupted sleep pattern. The MRI brain scan was initially normal, but by 16 years showed subtle global cerebral atrophy (particularly in the posterior regions) as well as cerebellar atrophy (Supporting Information Fig. S2). ^123^I‐FP‐CIT SPECT (DaTScan) at 17 years showed profound reduction in tracer uptake in the basal ganglia (Fig. 2D). CSF HVA and HVA:5‐HIAA ratio were reduced at ages 4 and 14 years. CSF‐HIAA levels were reduced at age 4 years (Fig. 3A). She showed an initial response to l‐dopa, but developed emotional lability at 5.5 mg/kg/d, leading to drug withdrawal. There was no clinical improvement observed with trihexyphenidyl or chloral hydrate. She had a modest response to pramipexole, with improved facial expression, reduced tremor, and increase in voluntary movements.

#### Family C

This 18‐year‐old girl is the third child of distantly related Latin American parents, with 2 healthy siblings. She initially presented with neonatal feeding difficulties and hypotonia. In infancy, she showed delay in attaining milestones and developed seizures characterized by staring episodes with loss of tone. She walked independently from 2 years, but by 10 years of age her gait deteriorated, leading to frequent falls, postural instability, and losing the ability to run. Over the next 4 years, she continued to deteriorate with worsening antecollis and bradykinesia (Video 6). She developed severe bulbar dysfunction with sialorrhea, dysarthria, and, dysphagia, leading to considerable weight loss. At 12 years, she developed generalized tonic‐clonic seizures and atypical absences, responsive to lamotrigine and zonisamide therapy. MRI demonstrated subtle generalized cerebral atrophy and CSF HVA was low (Fig. 3A). Her movement disorder responded to l‐dopa, with improved tremor, gait, and a reduction in drooling. A maximum of 200 mg/d was tolerated, but further increases led to intolerable drug‐induced dyskinesias. After 4 months of treatment, she developed aggressive behavior and received treatment with quetiapine. By 16 years, she became increasingly sensitive to l‐dopa, with peak‐dose agitation, restlessness, and dyskinesia. Lowering the dose improved side effects, and continued to provide motor benefit, although the effects wore off 2 to 3 hours after administration. *On*‐*off* phenomena were commonly reported, and in the *off* state, she was often akinetic and rigid. Introduction of trihexyphenidyl improved rigidity, but not immobility.

### Patient CSF and Fibroblast Analysis

CSF HPLC analysis of the *DNAJC6* patient cohort showed reduction in CSF‐HVA levels (*P* = 0.002) compared to controls (but not 5‐HIAA levels) in 3 patients (Fig. 3A,B). Patient fibroblasts showed reduced auxilin (*P* = 0.009) and a trend for increased GAK protein (*P* = 0.11; Fig. 3C). Patient CSF auxilin levels were even more significantly reduced (*P* = 0.0015; Fig. 3D). Notably, CSF‐GAK levels were significantly increased in patients (*P* = 0.0014; Fig. 3D). CSF immunoblotting studies showed that several key components of the dopaminergic synapse were significantly reduced, including tyrosine hydroxylase (TH; *P* = 0.0001), vesicular monoamine transporter (VMAT; *P* = 0.0002), dopamine transporter (DAT; *P* = 0.0003), and D2 receptor (D2R; *P* = 0.002; Fig. 3E).

## Discussion

Juvenile parkinsonism attributed to *DNAJC6* mutations has only recently been reported. Here, we report on a further 6 patients from three families, with two previously unreported homozygous nonsense mutations in *DNAJC6*. Moreover, our findings on ^123^I‐FP‐CIT SPECT (DaTScan) imaging, CSF analysis, and immunoblotting suggest downstream dyshomeostasis of auxilin, GAK, and dopaminergic proteins in *DNAJC6‐*related disease.

Our data confirms that all reported cases of juvenile‐onset *DNAJC6*‐parkinsonism have core clinical characteristics (Table 1), including (1) clinical presentation of progressive parkinsonism toward of the first decade (median, 10 years; range, 7–13), (2) significant neurological regression thereafter, and (3) loss of ambulation in mid‐adolescence.^12^, ^13^, ^14^, ^21^ In contrast to adult‐onset PD, childhood parkinsonian disorders rarely present with a “pure” parkinsonian phenotype, as illustrated by the classical primary pediatric monoamine neurotransmitter disorders.^20^ Similarly, in early‐onset *DNAJC6‐*related disease, parkinsonism is commonly present in tandem with a multitude of other clinical features, including dystonia, moderate learning difficulties, epilepsy, and neuropsychiatric features^12^, ^13^, ^14^, ^21^ (Table 1). Furthermore, many of our patients had evidence of bulbar dysfunction, gut dysmotility, and sleep disturbance. The majority of our patients showed limited response to l‐dopa and other standard therapies for parkinsonism‐dystonia. They experienced severe, often intolerable, side effects with dopaminergic agents, including *on*‐*off* phenomenon and severe dyskinesia, particularly at higher drug dosages.

^123^I‐FP‐CIT SPECT (DaTScan) was performed in 3 patients, demonstrating reduced tracer uptake in the basal ganglia, suggestive of impaired presynaptic dopamine uptake and striatonigral neurodegeneration. Postmortem studies have confirmed striatal dopamine deficiency in patients with parkinsonism.^22^, ^23^ Together, these observations suggest a neurodegenerative process in *DNAJC6* patients. MRI brain imaging further corroborates this hypothesis; the mild generalized cerebral and/or cerebellar atrophy in 4 patients suggests that *DNAJC6‐*related disorders may also be associated with neuronal loss in other regions of the central nervous system.

All 6 cases fit the juvenile phenotype associated with this gene, though more recently, *DNAJC6* mutations have been reported in early adult‐onset PD.^14^ Although there are a number of overlapping features (progressive parkinsonism, psychiatric features), affected patients presented later (range, 21–42 years) and seizures and cognitive decline are not reported.

Homozygous and compound heterozygous mutations in *DNAJC6* are predicted to result in loss of protein function. To date, splice‐site variants,^12^ large multiexonic deletions,^24^ truncating mutations,^13^ and missense mutations^14^ have been reported. All 6 patients in our cohort had nonsense mutations, predicted to cause nonsense‐mediated decay or premature protein truncation. Five of the 6 reported patients are from two consanguineous families originating from the same region in Pakistan, and all have the same nonsense mutation. SNP array confirmed a common haplotype at this disease locus for all affected children, suggesting a possible founder effect.

**Figure 4:**
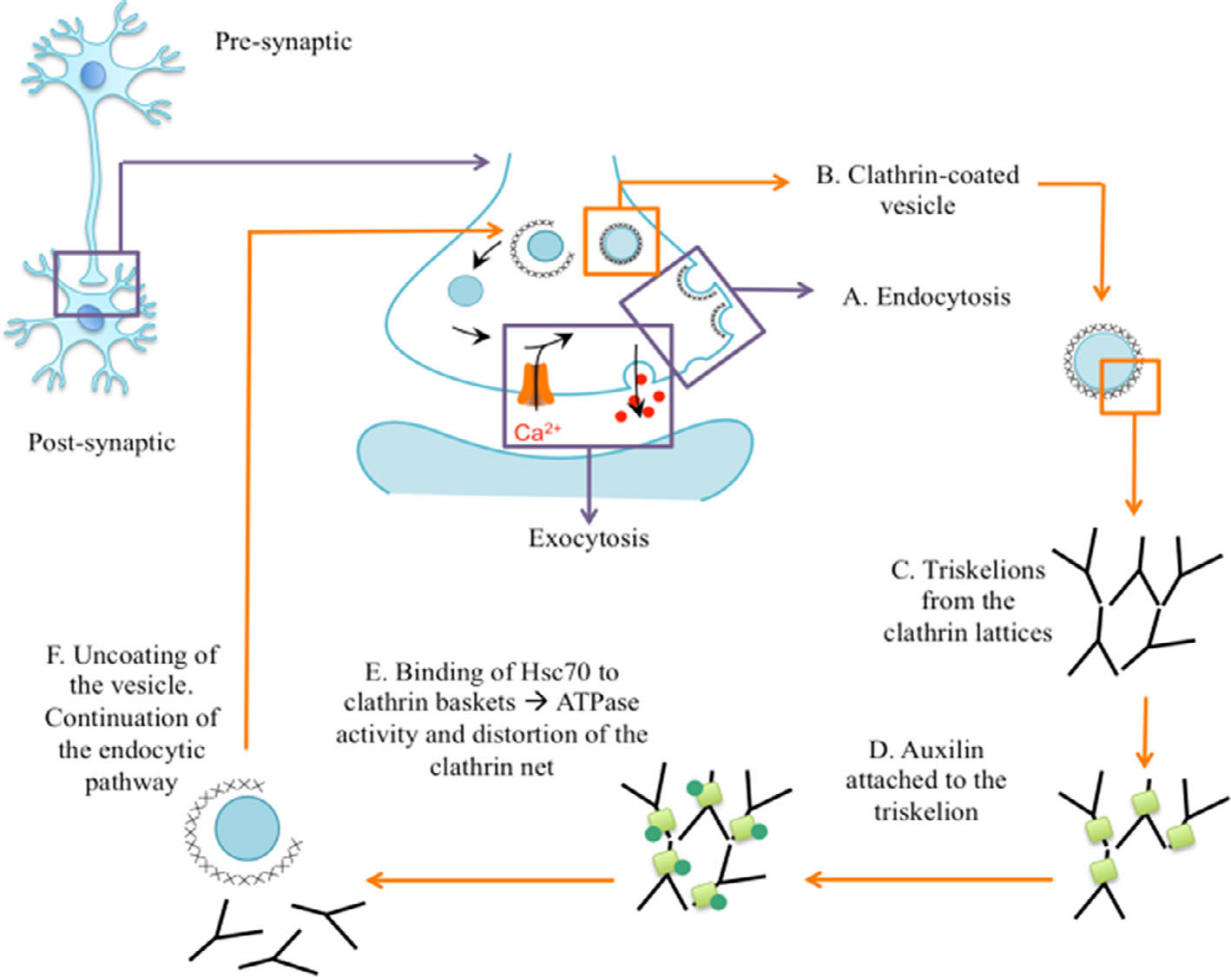
Schematic representation of *DNAJC6‐*encoded auxilin protein function. The role of auxilin in synaptic vesicle recycling and endocytic pathway. (**A**) A nascent clathrin‐coated pit is formed at the presynaptic membrane, followed by membrane invagination; (**B**) the pit develops into a clathrin‐coated vesicle, where the clathrin lattice consists of (**C**) clathrin triskelions formed by three crossed ankle regions. (**D**) Auxilin (pale green square) binds to an exposed domain in the heavy chain of the clathrin. (**E**) Auxilin facilitates a conformational change in clathrin that allows binding of Hsc70 and by ATPase‐mediated activity, and the clathrin lattice is disrupted and distorted, leading to (**F**) clathrin disassembly allowing subsequent delivery of cargo neurotransmitters to the membrane or other vesicle in the endocytic pathway. Hsc70, heat shock cognate protein 70. [Color figure can be viewed at wileyonlinelibrary.com]

*DNAJC6* encodes for auxilin, a neuronally expressed J‐chaperone protein involved in the uncoating of clathrin‐coated vesicles^25^, ^26^ (Fig. 4). Auxilin modifies the three‐dimensional conformation of heavy‐chain clathrin triskelions, leading to clathrin coat distortion, instability, and subsequent disassembly.^25^, ^26^ Neurotransmission involves rapid continuous recycling of synaptic vesicles through CME. Deficiency in auxilin ultimately results in impairment of synaptic vesicle recycling and impaired neurotransmission. Similarly, aberrant synaptic vesicular trafficking is also evident in other forms of early‐onset parkinsonism, including *LRRK2*,^27^, ^28^ *VMAT2*,^29^ and *SNCA*‐related disease.^30^ Clathrin‐mediated endocytosis is crucial for the regulation of developmental signaling pathways through internalization of receptors or ligands and is required for axon and dendrite outgrowth.^31^ Presence of developmental delay well before onset of parkinsonism in patients with *DNAJC6* mutations further corroborates the notion that auxilin is likely to have a central role in neurodevelopment, given its role in CME. In *Drosophila*, auxilin is crucial for Notch signaling, a developmental pathway that regulates neural stem‐cell proliferation, survival, renewal, and differentiation, as well as neuronal specification of dopaminergic neurons.^32^, ^33^, ^34^

To investigate the downstream effects of *DNAJC6* mutations, we studied auxilin and GAK protein levels in 2 patients using patient fibroblasts and CSF. Auxilin is a neuron‐specific protein, enriched in presynaptic terminals, whereas GAK is an ubiquituously expressed protein.^35^, ^36^ Auxilin and GAK are highly homologous proteins that both have the ability to bind clathrin and clathrin adaptor protein 2 in order to initiate clathrin uncoating of endocytosed vesicles.^37^ In the auxilin knockout mouse model, it is reported that upregulation of GAK can partially compensate for the loss of auxilin and decrease mortality.^35^ We therefore wished to determine whether a similar compensatory mechanism was evident in our patients. In our study, we observed that patient fibroblast auxilin protein levels were significantly reduced when compared to controls, as previously reported.^14^ We found that patient fibroblast GAK levels were slightly, but not significantly, increased, whereas patient CSF GAK protein levels were significantly increased. Our findings support upregulation of brain GAK levels in *DNAJC6* patients, partially compensating for auxilin reduction, as evident in the auxilin knockout mouse model.^35^

Diagnostic CSF neurotransmitter analysis revealed that levels of the stable dopamine metabolite (HVA) were either below the age‐related reference ranges or close to the lower limit of normal in our patients, indicating impaired dopamine turnover. Indeed, CSF‐HVA levels and HVA:5‐HIAA ratios were comparable to those observed in TH deficiency, an inherited dopamine synthesis defect associated with central dopamine deficiency.^20^ In order to determine how *DNAJC6* mutations may impact the dopaminergic system, we used patient CSF to analyze proteins involved in dopamine signaling and homeostasis. We observed that patient CSF had significantly reduced levels of VMAT, DAT, TH, and D2R when compared to controls. ^123^I‐FP‐CIT SPECT (DaTScan) imaging additionally provides in vivo evidence of impaired DAT function in *DNAJC6* patients. VMAT and DAT are both synaptic transporters recycled through clathrin‐mediated endocytosis.^38^, ^39^ The reduction in HVA associated with low VMAT/DAT protein levels may imply that the observed dopamine deficiency is associated with impaired clathrin‐mediated neurotransmitter recycling. D2R is also postulated to be internalized through clathrin‐mediated endocytosis.^38^, ^39^ Neurons internalize receptors to adjust excitability and degrade, resensitize, and recycle desensitized receptors.^38^, ^39^ *DNAJC6* mutations thus may affect D2R protein levels and normal postsynaptic function. It is likely that presynaptic D2R autoreceptor function will also be affected, leading to aberrant TH regulation.^40^

Overall, our findings suggest that the mechanisms governing *DNAJC6‐*associated parkinsonism are likely to be multifactorial. Another plausible explanation for the reduction in synaptic protein levels may be as a result of neurodegeneration secondary to defective chaperone function. Auxilin and other J‐chaperone proteins play a crucial role in regulating the folding and conformational change of proteins to maintain integrity in the neuron.^41^, ^42^ Indeed, in the auxilin knockout mouse model, there is sequestration of clathrin cages in the cerebellum.^35^ With impaired auxilin function, a cumulative effect of sequestered misfolded proteins and accumulation of clathrin coat components in assembled coats and cages may lead to apoptotic cascades and neurodegeneration. There is growing interest in the role of such chaperone proteins in human disease and mutations in eight distinct J proteins (*DNAJB2*, *DNAJB6*, *DNAJC5*, *DNAJC6*, *DNAJC12*, *DNAJC13*, *DNAJC19*, and *DNAJC29*) have been described.^43^, ^44^ Future research into such “chaperonopathies” may provide further insights into neurodegenerative disorders.

In conclusion, we report on a cohort of patients with previously unreported *DNAJC6* mutations associated with early neurodevelopmental delay, juvenile parkinsonism, and neurological regression in the second decade of life. We further demonstrate disturbance of dopamine homeostasis in patient‐derived CSF and report on a possible GAK‐mediated compensatory mechanism for auxilin deficiency. Mutations in *DNAJC6* are rare, but a likely under‐recognized cause of parkinsonism‐dystonia in infants and children. Elucidating the genetic diagnosis has important implications for families given that early diagnosis negates the need for extensive invasive investigations, facilitates treatment strategies, and aids genetic counseling for future pregnancies. The early clinical features and CSF neurotransmitter signature observed in our patients can mimic primary neurotransmitter disorders, and *DNAJC6* mutations should thus be considered as a differential diagnosis. We observed reduced auxilin and increased GAK protein levels, suggesting a possible compensatory role for GAK in this condition. Study of CSF synaptic proteins suggest downstream effects on dopamine synthesis, recycling, homeostasis, and signaling that may result from a combination of primary auxilin deficiency and neurodegeneration. Abnormal synaptic vesicle dynamics are increasingly recognized as a disease mechanism in neurodegenerative parkinsonian disorders, and future research into elucidating the pathogenesis of such conditions will no doubt assist the development of novel targeted treatments.

## Author Roles

(1) Research Project: A. Conception and Design; B. Acquisition of Data; C. Analysis and Interpretation of Data; (2) Manuscript: A. Writing of the First Draft, B. Review and Critique; (3) Other: A. Statistical Analysis (for Figure 3); B. Drafting of Figures; C. CSF Neurotransmitter Analysis and Interpretation.

J.N.: 1A, 1B, 1C, 2A, 3A

E.C.‐S.: 1A, 1B, 1C, 2A, 3B

L.A.: 1B, 1C, 2A, 3B

P.T.: 1B, 1C

K.M.: 1B, 1C

S.S.: 1B, 1C

K.M.G.: 1B

S.J.R.H.: 3C

S.P.: 3C

L.B.: 1B, 1C

B.C.: 1B, 1C

J.C.: 1B, 1C

K.R.: 1B, 1C

H.C.: 1B, 1C

S.J.: 1B, 1C

R.J.: 1B, 1C

D.H.: 1B, 1C

À.G.‐C.: 1B, 1C

D.G.: 1B, 1C

F.L.R.: 1B, 1C

B.P.‐D.: 1B, 1C

C.D.G.: 1B, 1C

T.S.P.: 1B, 1C

E.M.: 1A, 1B, 1C, 2A

M.A.K.: 1A, 1B, 1C, 2A

## Financial Disclosures

Nothing to report.

## Supporting information

**Video 1** Patient A‐III:1. She is now 20 years old. Examination in the *off* state, >3 hours after taking medication. She is hypomimic, has resting tremor more evident in her right foot, and has contractures and deformities in both hands and feet. She is markedly bradykinetic, and this is evident when she tries to smile.

**Video 2** Patient A‐III:4. He is now almost 12 years old. Tremor is evident in resting state. Bradykinesia is evident when grasping. Mild hypomimia is present. There is no evidence of oculomotor abnormalities or upgaze palsy. He regularly touches his earlobe as a sensory trick attempting to control his tremor. Rigidity and striatal toe are evident when walking. Improvement of dystonia is seen upon running. Dystonic posturing (especially in the right hand) is also present when ambulant. He has difficulties in maintaining normal balance when he is standing.

**Video 3** Patient A‐III:5. He has recently turned 10 years old. He has evidence of resting tremor and bradykinesia. No abnormal eye movements are present. The gait is mildly affected as he can run and walk, but has dystonic posturing of both hands. Improvement of dystonic posturing is seen upon running. He has trouble in trying to walk on a line, showing difficulties in maintaining balance.

**Video 4** Patient B‐IV:2. She is now 27 years old. She has significant disability and is completely wheelchair bound. She is very hypomimic and has poor head control. She is able to understand simple commands, but she is extremely hypokinetic. She exhibits some sudden muscle contractions from both arms and legs. She has fixed distal deformities in her hands and feet, with dystonic postures. No signs of spasticity are present.

**Video 5** Patient B‐IV:4. She is now 17 years old. She has poor head control. She is also very hypomimic. She can understand commands. She exhibits a clear tremor. She has episodic whole‐body shaking, almost always related to new situations when she is anxious or nervous. These episodes are short and rarely last for more than 1 minute. Bradykinesia is evident on finger‐finger testing. Cogwheel rigidity is more evident than in her older sister, B‐IV:2. Distal tremor from her left foot is shown.

**Video 6** Patient C. Segment 1 demonstrates examination in the *off* state at age 14. There is moderate facial masking with prominent lower facial dystonia. She has moderate‐to‐severe dysarthria. Hand grasping, finger tapping, and foot tapping is moderately impaired bilaterally, left slightly greater than right. There is moderate generalized bradykinesia. Her gait is characterized by mildly reduced stride length, reduced bilateral arm swing, some festination, and a tendency to fall forward. Pull test is negative. Segment 2 illustrates examination in the *off* state at age 17, which shows progression from segment 1, taken 3 years earlier. She has marked anterocollis with more prominent lower facial dystonia. She is almost mute. She can follow some simple commands, but her movements are markedly bradykinetic with long latency to respond. She has prominent drooling. Finger‐to‐nose testing, hand grasping, and foot tapping reveal marked bradykinesia bilaterally. She requires assistance from one person to stand up from a chair and has a tendency to fall backward. She can take only a few steps very slowly, requiring assistance from other people.

**FIG. S1** Brain MRI in patient A‐III:1. Patient A‐III:1: axial T_2_ and coronal FLAIR images at ages 12 and 19 years showing progressive right frontoparietal and perisylvian (black star) atrophy over time. There is also evidence of right cerebellar atrophy between the two studies (white arrow). FLAIR, fluid‐attenuated inversion recovery.

**FIG. S2** Brain MRI in Patient B‐IV:4. Patient B‐IV:4: Sagittal T_1_ and axial T_2_ MR images at age 12 and 16 years show a subtle, but definite, global brain parenchymal volume loss over time, particularly in the posterior aspects of the cerebral hemispheres. The occipital horns of the lateral ventricles are, as a result, more dilated over time. There is also subtle cerebellar atrophy between the two studies as shown on the sagittal T_1_ images.
