# Supplementary figures and images for: DNAJC6 Mutations Disrupt Dopamine Homeostasis in Juvenile Parkinsonism‐Dystonia
Source: Mov Disord. 2020 May 30;35(8):1357–68. doi: 10.1002/mds.28063 (PMC8425408; doi:10.1002/mds.28063)

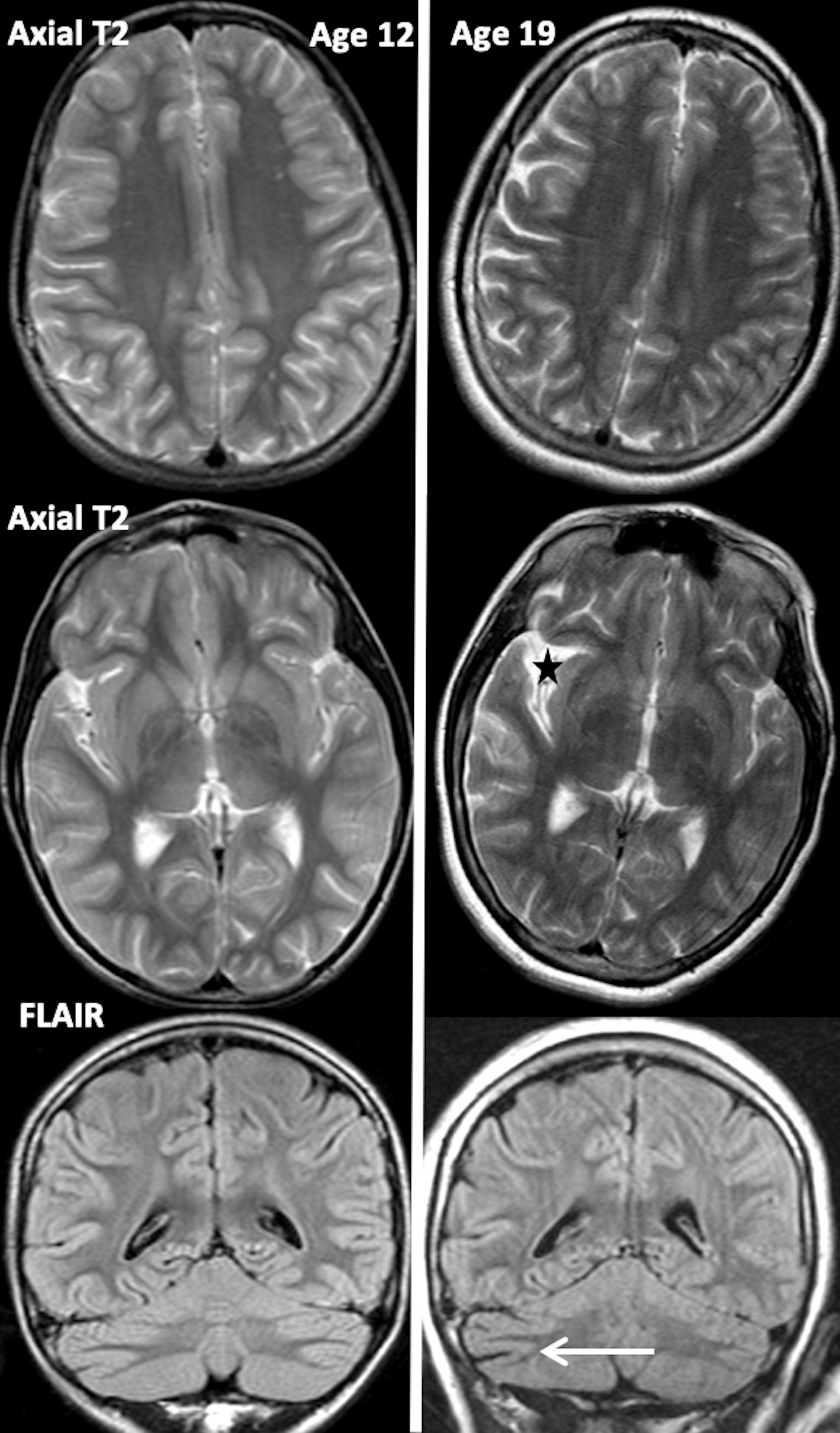

Supplement: Supplementary file 7 — FIG. S1 Brain MRI in patient A‐III:1. Patient A‐III:1: axial T2 and coronal FLAIR images at ages 12 and 19 years showing progressive right frontoparietal and perisylvian (black star) atrophy over time. There is also evidence of right cerebellar atrophy between the two studies (white arrow). FLAIR, fluid‐attenuated inversion recovery. [file MDS-35-1357-s001.tif]

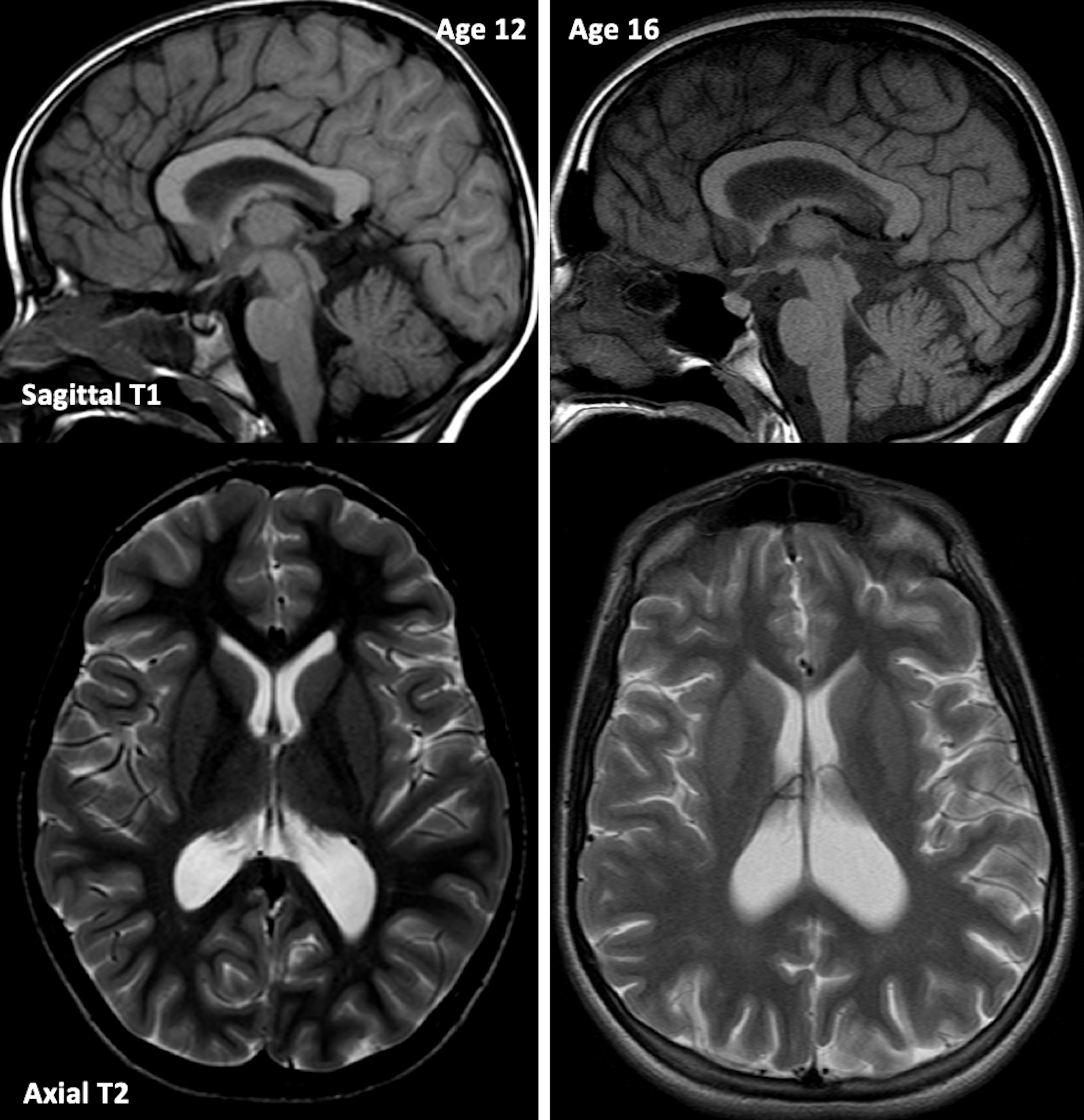

Supplement: Supplementary file 8 — FIG. S2 Brain MRI in Patient B‐IV:4. Patient B‐IV:4: Sagittal T1 and axial T2 MR images at age 12 and 16 years show a subtle, but definite, global brain parenchymal volume loss over time, particularly in the posterior aspects of the cerebral hemispheres. The occipital horns of the lateral ventricles are, as a result, more dilated over time. There is also subtle cerebellar atrophy between the two studies as shown on the sagittal T1 images. [file MDS-35-1357-s002.tif]
